# Conjunction of triboelectric nanogenerator with induction coils as wireless power sources and self-powered wireless sensors

**DOI:** 10.1038/s41467-019-13653-w

**Published:** 2020-01-02

**Authors:** Chi Zhang, Jinkai Chen, Weipeng Xuan, Shuyi Huang, Bin You, Wenjun Li, Lingling Sun, Hao Jin, Xiaozhi Wang, Shurong Dong, Jikui Luo, A. J. Flewitt, Zhong Lin Wang

**Affiliations:** 1 Ministry of Education Key Labaratory of RF Circuits and Systems, College of Electronics & Information Hangzhou Dianzi University, Hangzhou, China; 2 Key Labaratory of Advanced Micro/Nano Electronic Devices & Smart Systems of Zhejiang, College of Information Science & Electronic Engineering, Zhejiang University, Hangzhou, China; 3 Institute of Renewable Energy & Environmental Technology, Bolton University, Deane Road, Bolton, BL3 5AB UK; 4 Electrical Engineering Division, University of Cambridge, JJ Thomson Avenue, Cambridge, CB3 0FA UK; 5 Beijing Institute of Nanoenergy and Nanosystems, Chinese Academy of Sciences, Beijing, 100083 China

**Keywords:** Energy science and technology, Engineering, Nanoscience and technology

## Abstract

Here we demonstrate a magnetic resonance coupling based wireless triboelectric nanogenerator (TENG) and fully self-powered wireless sensors. By integrating a microswitch and an inductor with the TENG, the pulsed voltage output is converted into a sinusoidal voltage signal with a fixed frequency. This can be transmitted wirelessly from the transmit coil to the resonant-coupled receiver coil with an efficiency of 73% for a 5 cm distance between the two coils (10 cm diameter). Analytic models of the oscillating and coupled voltage signals for the wireless energy transfer are developed, showing excellent agreement with the experimental results. A TENG of 40 × 50 mm^2^ can wirelessly light up 70 LEDs or charge up a 15 μF capacitor to 12.5 V in ~90 s. The system is further utilized for two types of fully self-powered wireless chipless sensors with no microelectronic components. The technologies demonstrate an innovative strategy for a wireless ‘green’ power source and sensing.

## Abstract (web summary)

Renewable energy based wireless power transfer is challenging. Here the authors show a resonant-coupled triboelectric nanogenerator as a wireless power source and have further utilized this system to explore two fully self-powered wireless chipless sensors.

## Introduction

“Green” energy harvesting technologies have attracted great attention owing to their significant potential in tackling the crises of energy shortage and climate change by providing sustainable energy for the rapidly increasing number of personal electronic devices, sensors, and other components that comprise the Internet of Things (IoT), wearable/portable electronics^1–4^. Various technologies have been explored, such as thermoelectric, piezoelectric, and electrostatic nano/micro-generators with output power density from a few of μW cm^−^^2^ to several tens of mW cm^−2^ (refs. ^5–9^). The triboelectric nanogenerator (TENG) is a recently invented energy harvesting strategy based on the combination of triboelectrification and electrostatic induction effects, and has the merits of being low cost, high power output and energy conversion efficiency with wide choices of tribomaterials^10,11^. The instantaneous output power density of TENGs has increased steadily from a few μW cm^−^^2^ initially to ~32 mW cm^−^^2^ recently^11–14^. With this level of power density, it is possible to use TENGs as an energy source for applications, such as self-powered sensing, wearable electronics, and IoT devices^15–18^. However, these typically involve multiple energy conversion processes: mechanical energy to electricity conversion, storage and voltage regulation to power electronics. The energy conversion at each stage results in energy loss, thus significantly reducing total energy efficiency^19–21^. Therefore, a technology that could directly use the harvested energy as wireless sensing signals would be a significant advance for the development of IoT, wireless sensor networks etc.

Wireless power transmission (WPT) has received great attention owing to consumer demands and scientific challenges^22–24^. In most cases, WPT refers to the wireless transmission of energy by means of electromagnetic fields although many studies have also shown that ultrasonic waves, mechanical waves etc. can be utilized to wirelessly transmit energy^25,26^. The wireless energy transmission systems based on electromagnetic field can be divided into the near-field WPT and far-field WPT. The former includes inductive coupling^27^, resonant inductive coupling^22,28^, and capacitive coupling^23^ methods. The transmission range of these techniques is relatively short, and strongly depends on the size and shape of the “antenna” used. The far-field WPT technologies are typically based on microwaves^29^ and lasers^30^ etc. and are able to transmit power up to a few kilometers. Among the WPT technologies, magnetic resonance-based WPT has the advantages of long operation distance up to several meters, high power transmission (up to kilowatts) and high transmission efficiency^24^, and has already been commercialized for applications. At present, most TENGs have energy extracted through wire connections. If the energy output of TENGs could be collected wirelessly, then their applications could be broadly extended, particularly for self-powered hash environmental sensing/monitoring and implants^31–33^. Thus, it would be tremendously useful to develop TENGs with WPT capability.

A recently reported wireless TENG was based on the transmission of the pulsed voltage output generated by the TENG directly^23^. The pulsed voltage was transmitted through a wire antenna without modulation, the voltage signal received by the receiver antenna decayed quickly with the increase of antennas distance. Wang et al. reported a wireless energy harvesting based on the Maxwell displacement current with the distance limited to a few centimetres^34,35^. Overall, in contrast to the significant progress related to TENGs in developing new tribomaterials, structures, storage methods, and applications, the study on wireless TENGs is very limited.

Here, we propose a magnetic resonance-based wireless triboelectric nanogenerator (MR-WTENG). By integrating an inductor coil with a capacitive-type TENG and a synchronized microswitch, the pulsed voltage output of the TENG is converted into a sinusoidal voltage with a fixed resonant frequency. These a.c. signals are transmitted out wirelessly through a primary inductor coil and are received by a secondary inductor coil at a distance. Results show that the energy transmission efficiency is ~73% for a distance of 5 cm between the coils with a diameter of 10 cm, though it decreases quickly with increase in coil distance. An MR-WTENG with a size of 40 × 50 mm^2^ can wirelessly light up 70 blue light-emitting diodes (LEDs) or charge a 15 µF capacitor up to 12.5 V in ~90 s. Furthermore, the system is directly utilized for fully self-powered wireless sensing up to a distance of 2 m.

## Results

### System structure of MR-WTENG

**Fig. 1:**
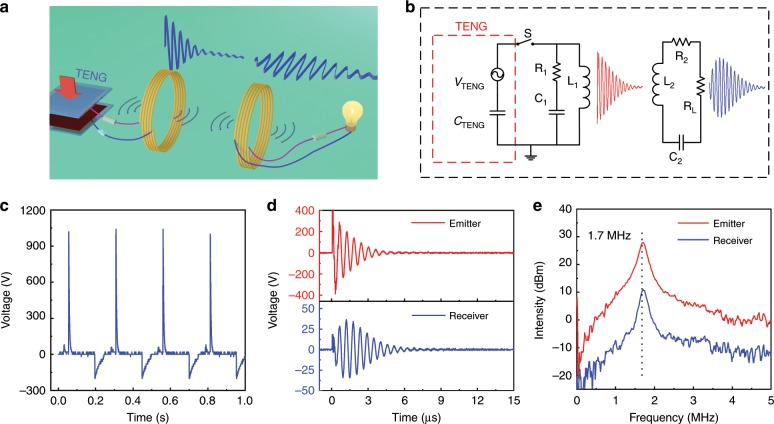
Magnetic resonance coupled wireless TENG. **a** The schematic and **b** the equivalent circuit of the magnetic resonance-coupled wireless energy transmission system. **c** The typical voltage output of the contact-separation mode PA6/PDMS TENG with the microswitch under cyclic pressing. **d** The oscillating voltage signals and **e** their fast Fourier transformation spectra of the emitter and receiver modules of the MR-WTENG system. The working condition of the TENG for **c**, **d**, **e** is 50 N contact force, 4 Hz contact frequency, and 6 mm spacer distance between the two triboplates, and the transmission distance between the two coils is 5 cm.

Figure 1a, b are the schematic structure of the proposed MR-WTENG. A contact-separation mode TENG was used in this work, though other modes of TENGs (sliding, single electrode, and freestanding modes)^36,37^ are also suitable for the proposed MR-WTENG. The TENG consists of a polyamide-6 (PA6, Rhodia Ltd.) positive tribomaterial and a polydimethylsiloxane (PDMS, 184 Silicone Elastomer, Dow corning Co. Ltd.) negative tribomaterial. Figure 1c is the voltage output of the TENG with integrated microswitch with a 100 M Ω load (50 N contact force, 4 Hz contact frequency, and 6 mm separation). The detailed performance of the PA6/PDMS TENG under various conditions is shown in Supplementary Fig. 1 in Supplementary Note 1.

The schematic of the MR-WTENG and its equivalent circuit are shown in Fig. 1a, b. These show a TENG integrated with a microswitch and an *L*_1_*C*_1_ resonant circuit as the transmitter. The receiving module is composed of an inductor coil *L*_2_ and a tuning capacitor *C*_2_ in series connection. *R*_*L*_ is the load resistance, and *R*_1_ and *R*_2_ are the parasitic resistances of the inductor and wires. An oscillating signal with attenuating amplitude will be generated, when the pulsed voltage (energy) of the TENG is coupled to the *L*_1_*C*_1_ circuit, as shown in Fig. 1d. To establish a stronger electromagnetic coupling between the transmitter and receiver, the resonant frequency of the *L*_2_*C*_2_ receiver circuit is tuned to the same frequency as the transmitter by varying the value of *C*_2_. The coils of the transmitter and receiver are aligned coaxially to form a strongly coupled system, as such a high-efficiency WPT is established. Figure 1e is the fast Fourier transform (FFT) spectrum of the transmitted and received voltage signals respectively, with a 5 cm transmission distance, h, between the coils (hereafter called the coil distance, h,).

### Theoretical analysis of MR-WTENG

The output impedance of TENGs is usually very high^38,39^, in the range of 10–1000 MΩ, while the impedance of the *C*_1_ and *L*_1_ resonant circuit is very small, typically <100 Ω. If the TENG is directly connected to the LC circuit, the impedances of the TENG and the LC resonant circuit are highly mismatched, resulting in a very small voltage output on the coil, and most of the energy from the TENG cannot be transferred. Our previous work^40,41^ showed that by using a synchronized microswitch in a series connection to the TENG, the output impedance of the TENG could be significantly reduced, resulting in an optimal impedance match to the load, and the output power coupled to the LC circuit can be enhanced significantly. A single-contact microswitch was used in the previous work, which only closes at the contact state^40^. In this study, a bi-contact microswitch was used which makes it possible to collect both the positive and negative voltage pulses generated by the TENG. The microswitch is closed when the two triboplates are either in contact or at maximum separation. Once the microswitch is closed, a current pulse is injected into the LC circuit, and an oscillating signal is generated with the amplitude attenuating due to the finite pulse energy input with the oscillating frequency determined by the value of *L*_1_, *C*_1_, and *C*_TENG_. The schematic structure of the contact-separation TENG and its basic analysis are shown in Supplementary Fig. 2 of Supplementary Note 2. The operating principle of the bi-contact microswitch of the MR-WTENG and analysis of the two oscillating signal generation are shown in Supplementary Fig. 3 of Supplementary Note 3.

By varying the value of *C*_2_, the resonant frequency of the receiver can be tuned to be the same as that of the transmitter. When the two coils are aligned coaxially at a suitable distance, a magnetic resonance coupling is established and power will be transmitted wirelessly from the TENG to the receiver efficiently. The theoretical analysis of the power transfer of the MR-WTENG is based on analysing the case when the transmitter is powered by the positive pulse of the TENG output for simplicity, and the analysis of the negative pulse would be the same with the only difference being the value of *C*_TENG_. In the analysis, the input for the LC resonant circuit is a Dirac delta function, *U*_0_*δ*(*t*). Assume *u*_1_ the voltage crosses the inductor *L*_1_ and *u*_2_ the voltage crosses the load *R*_*L*_, then *u*_1_ and *u*_2_ can be obtained as follow,

1 $$L_1\frac{\mathrm{d}^{2}i_{1}}{\mathrm{d}t^{2}} - M\frac{\mathrm{d}^{2}i_{2}}{\mathrm{d}t^{2}} + R_{1}\frac{\mathrm{d}i_{1}}{\mathrm{d}t} + \frac{i_{1}}{C_{1} + C_{\mathrm{TENG}}} = U_{0}\delta (t)$$

2 $$L_{2}\frac{\mathrm{d}^{2}i_{2}}{\mathrm{d}t^{2}} - M\frac{\mathrm{d}^{2}i_{1}}{\mathrm{d}t^{2}} + \left( R_{2} + R_{L} \right)\frac{\mathrm{d}i_{2}}{\mathrm{d}t} + \frac{i_{2}}{C_{2}} = 0$$

3 $$u_{1} = L_{1}\frac{\mathrm{d}i_{1}}{\mathrm{d}t}$$

4 $$u_2 = R_Li_2$$

here *i*_1_ and *i*_2_ are the currents in coil 1 and coil 2, respectively, *M* is the mutual inductance between the two coils, *C*_TENG_ is the capacitance of the TENG with the expression shown in Eq. (1) in Supplementary Note 2, and *U*_0_ is the initial pulse voltage of the *C*_1_*L*_1_ circuit. The analytical solution of the attenuating transmission signal can be obtained by using the Laplace transform method. Specific solutions of the differential equations are described in Supplementary Note 4 and the solutions of *u*_*1*_ and *u*_*2*_ are expressed as follows:

5 $$u_1(t) = k_1e^{\alpha _1t}\sin (\omega _1t + \varphi _1) + k_2e^{\alpha _2t}\sin (\omega _2t + \varphi _2)$$

6 $$u_2(t) = k_3e^{\alpha _1t}\sin(\omega _1t + \varphi _3) + k_4e^{\alpha _2t}\sin (\omega _2t + \varphi _4)$$

It is clear that both the transmitted and received voltages contain two oscillating components with different angular frequencies (*ω*_1_, *ω*_2_), and both voltages decay with time. *k*, *α*, and *φ* represent the amplitude, attenuation coefficient, and initial phase of the corresponding signal, respectively. The solutions of the undetermined coefficients in Eqs. (5) and (6) are very complicated, but numerical methods can be used to find specific values. With the parameters shown in Table 1 for the resonant-coupled state, all the coefficients can be solved. Supplementary Fig. 4 in Supplementary Note 4 shows the relationships between the coefficients and *C*_2_.

**Table 1:** Parameters of MR-WTENG system at the resonant-coupled state.

Diameter of coil 1, d_1_	10 cm
Diameter of coil 2, d_2_	10 cm
Transmission distance, h	5 cm
Inductance, *L*_1_	62.5 µH
Inductance, *L*_2_	62.5 µH
Resistance of inductor, *R*_1_	11 Ω
Resistance of inductor, *R*_2_	11 Ω
Load, *R*_*L*_	150 Ω
Capacitance, *C*_1_+*C*_TENG_	140 pF
Capacitance, *C*_2_	140 pF
Mutual inductance, *M*	7.5 µH
Coupling coefficient, *k*	0.12

The amplitudes of the two signal components of Eqs. (5) and (6) are a maximum at the resonant-coupled state (*L*_1_ = 62.55 μH, *L*_2_ = 62.55 μH, *C*_2_ = 140 pF = *C*_1_ + *C*_TENG_), where *ω*_1_ has the smallest difference from *ω*_2_ as shown in Supplementary Fig. 4c. Supplementary Figure 4b shows that the initial phase difference between the two signal components is *π* at the resonant state because of the existence of the inductor and capacitor, thus at the beginning of oscillation, the two signal components cancel each other. Supplementary Figure 4d shows that the absolute values of the attenuation coefficients of the two signals are equal with a relatively large value of −0.75 × 10^6^ at the resonance, which means that at the resonant-coupled state, both the transmitting and receiving signals will decay faster compared to that of nonresonant-coupled state. The calculated waveforms of individual components and the received signal in resonance are shown in Supplementary Fig. 4e, f respectively, which is consistent with the experimental results in Fig. 1d.

### Energy transfer control using the C_2_ capacitance

**Fig. 2:**
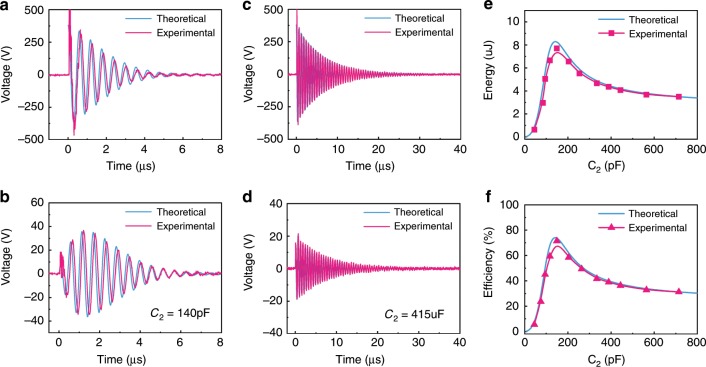
Influence of capacitance *C*_2_ on energy transmission. **a** The transmitted and **b** the received voltage waveforms with the system working at the resonant-coupled state. **c** The transmitted and **d** the received voltage waveforms with the system working at a nonresonant-coupled state. **e** The transferred energy value and **f** the energy transmission efficiency of the MR-WTENG system as a function of *C*_2_. All of the experiments and theoretical analyses were conducted using the conditions shown in Table 1 with *C*_2_ as a variable.

**Fig. 3:**
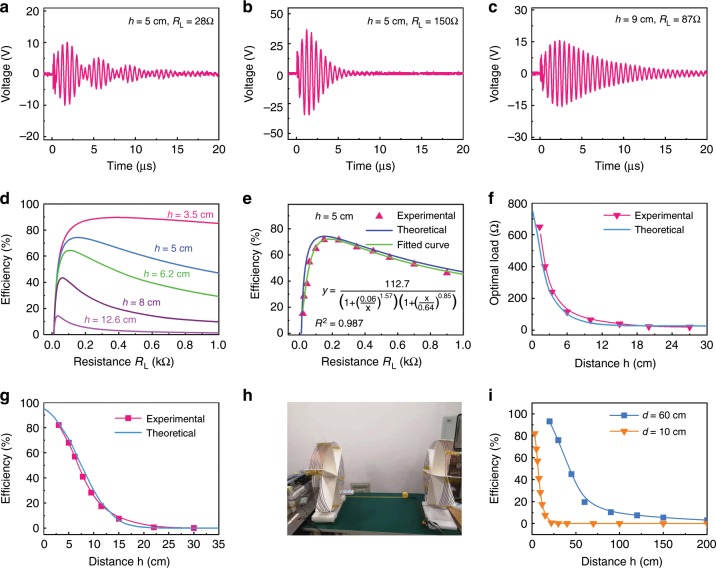
Effects of load, coil distance, and coil diameter on energy transmission. **a**, **b**, **c** are the received voltage waveforms at different distances between the two coils and different load resistances. **d** The theoretical calculated energy transmission efficiency of different transmission distances, as a function of load resistance. **e** The theoretical and experimental energy transmission efficiency (with the distance between the two coils set at 5 cm), as a function of load resistance. **f** The optimal load resistance and **g** the energy transmission efficiency of the MR-WTENG system, as a function of distance between the two coils. Here, the diameters of the coils are 10 cm, with an inductance of 62.5 μH. **h** The photo of the coils with a diameter of 60 cm for the MR-WTENG system. **i** The comparison of the transmission efficiency under various distances between the coils with different coil diameters of 10 cm and 60 cm, respectively.

On the basis of the theoretical analysis, a MR-WTENG system was designed and assessed. The parameters used are summarized in Table 1 for the resonant-coupled state. Figure 2a, b compare the experimental and theoretical results of the transmitted and received voltage waveforms respectively, with the system working at the resonant-coupled state (in this case, *C*_2_ = 140 pF). The maximum *V*_PP_ of the transmitted voltage is ~800 V, while that of the received voltage is *V*_PP_ ≈ 80 V. Figure 2c, d are the results of the transmitted and received voltage signals respectively, with the system working at a nonresonant-coupled state (in this case, *C*_2_ = 415 pF). The received signal is only *V*_PP_ ≈ 30 V with the same transmitted voltage of 800 V, which is a significant reduction compared to the resonant-coupled case. For both the resonant-coupled and nonresonant-coupled states, the experimental results are in good agreement with the theoretical ones, indicating the developed model of wireless energy transmission for pulse energy input is accurate and valid (Supplementary Note 5–7). We define *µ* the ratio of the coupling distance, *h*, to the coil radius, *µ* = *h*/*r*, which is fixed at µ = 1 for both Figs. 2 and 3.

It can be noticed there is an abnormal jittering signal at beginning of the received oscillating signals, as shown in Fig. 2a and b, that are caused by high frequency components of parasitic parameters of the receiver. However, the signal amplitude is very small with the energy transmission <1% of the total transmitted energy, so that it is ignored in the analysis. A detailed discussion on this is provided in Supplementary Fig. 8 of Supplementary Note 8.

There are three distinct differences between the resonant-coupled and nonresonant-coupled cases. Firstly, at the nonresonant-coupled state, the oscillating voltage lasts longer, up to 70 cycles which is ~40 μs as determined by the attenuation factor, before it attenuates to zero. Whereas the resonant-coupled transmission only oscillates for ~10 cycles and attenuates to zero much more quickly in ~8 μs; this is consistent with the results predicted in Supplementary Fig. 4d which shows higher attenuation coefficients for both the transmitter and receiver at the resonant state (*C*_2_ = 140 pF). This phenomenon can be explained because wireless energy transfer is slow and inefficient in the nonresonant-coupled case; the energy remains in the transmitter circuit and continues to oscillate. Secondly, in the resonant-coupled state, the received signal has a larger amplitude than that of the nonresonant-coupled state, implying an efficient energy transfer by the resonant-coupled circuit. Thirdly, the received oscillating voltage of the resonant-coupled circuit is not a monotonic attenuating signal, but a pear shaped one as predicted by the theoretical analysis as shown in Supplementary Fig. 4f.

The total energy received by the transmitting circuit from the TENG can be calculated from *W* = (*C*_1_ + *C*_TENG_)*U*_0_^2^/2 to be 11.2 μJ, while the energy transmitted to the receiver is equal to the energy consumed by the load resistance, *R*_*L*_, and can be calculated by integrating the power consumed by the load over time. The influence of capacitor *C*_2_ on the transferred energy and energy transfer efficiency between the transmitter and receiver were investigated experimentally and theoretically with the load resistance fixed at *R*_*L*_ = 150 Ω, with the results shown in Fig. 2e, f, respectively. Both increase with capacitance initially, reach a maximum at the resonant state, and then decrease with the increase of capacitance. Figure 2e is the energy received by the receiver circuit as a function of *C*_2_, clearly showing the maximum energy transfer is achieved at *C*_2_ = *C*_1_ + *C*_TENG_ = 140 pF (the resonant-coupled state). Similarly, the energy transmission efficiency also reaches its maximum at *C*_2_ = 140 pF. At *C*_2_ = 140 pF, the energy consumed by the load (*R*_*L*_ = 150 Ω) is 8.18 μJ, compared with 4.12 μJ for the case of *C*_2_ = 415 pF which is not a resonant-coupled state, representing the energy harvesting efficiencies of 73% and 36.8%, respectively. Therefore, it is clear that the resonant-coupled system can significantly improve the energy transfer efficiency.

### Energy transfer controlled by a load

For the magnetic resonance WPT, it is known that the load would affect the energy transmission efficiency, and an impedance matching network is normally used to improve the efficiency.^42^ For our system, the load resistance was also found to influence the energy transfer efficiency as shown in Fig. 3. The received energy increases with the load resistance initially, reaches a maximum, and then decreases with increase of resistance with the optimal value of *R*_*L*_ depending on the coil distance (Fig. 3d, e). For the resonant-coupled case with the conditions summarized in Table 1, the energy transmission efficiency is 73% for the 150 Ω optimal resistance, and becomes 43.3% for a 28 Ω nonoptimal resistance.

Figure 3a, b shows the measured oscillating voltages with *R*_*L*_ = 28 Ω and 150 Ω, respectively. For *R*_*L*_ = 28 Ω, the waveform of Fig. 3a of the received voltage has multiple envelops due to the nonoptimal load and is different from that in Fig. 3b with the optimal load *R*_*L*_ = 150 Ω. Supplementary Fig. 5a, b shows the theoretical analysis for the influence of *R*_*L*_ on the voltage signal. It is clear that *R*_*L*_ influences the parameters *k*, *ω*, and *α* of the received signal; at nonoptimal load, the received voltage shows multiple envelops. This is because the received waveform contains two oscillating components with frequencies slightly different from each other (Supplementary Fig. 5a) and the signal decays slower at small *R*_*L*_ = 28 Ω. Superimposing the two oscillating components results in a multiple envelop signal as shown in Supplementary Fig. 5b, in agreement with the experimental observation (Fig. 3a). From the above results, it can be concluded that the load resistance not only consumes the energy, but also affects the coupling between the transmitter and receiver. Therefore, the load resistance should be chosen appropriately, based on the real conditions, in order to obtain high efficiency energy transfer.

### Energy transfer controlled by coil distance

The energy transmission efficiency of a traditional resonant-coupled WPT system (powered by a constant sinusoidal voltage source) is known to decrease with the increase of coupling distance. This was found to be true for our MR-WTENG system with a pulsed voltage source as well. When the two coils are aligned coaxially as shown in Supplementary Fig. 6a mutual induction is established, and the greater the mutual inductance, the better the energy transmission efficiency of the system. The mutual inductance, *M*, is determined by the radius of each coil, the coil distance, and the turn number of the coils. The coupling coefficient is given as,

7 $$K =\frac{{\mathrm{M}}}{{\sqrt {L_1 \cdot L_2} }}$$

which reflects the tightness of the coupling of two coils. For this analysis, the diameter and coil turns were kept the same. Only the distance was varied. The results indicate that the coupling coefficient is dependent on the coil distance, more specifically on the coil distance to coil radius ratio, *µ*. A detailed numerical analysis of the mutual inductance *M* as a function of the coil distance and *µ*, and the coupling coefficient are shown in Supplementary Fig. 7. The results show that the mutual inductance is at maximum when the coils have the same radius; and higher mutual inductance can be achieved by using a shorter coil distance. The coupling coefficient decreases with increase in coil distance.

The experimental coupling coefficients between the two coils were found to decrease rapidly with increase in the coil distance, with the result shown in Supplementary Fig. 7d, showing excellent agreement between the theoretical calculation and experiments. The results indicate that the amplitude of the voltage signals and the energy efficiency for the receiver vary significantly with coupling distance.

Figure 3b, c are typical received voltage waveforms at the resonant state at different coil distances with the loads being tuned at the optimal values. The voltage decreases from ~80 V to ~30 V, when the coil distance increases from 5 to 9 cm. The dependence of the optimal load and transfer efficiency on coil distance are shown in Fig. 3f, g. The optimal load resistance decreases when the coil distance increases. For high efficiency energy transfer, the load resistance for each coil distance needs to be optimized.

Table 2 is the performance summary of the MR-WTENG at different coil distances. The other parameters of the system are the same as those in Table 1 except that the load resistance is varied to maximise the output voltage at each coil distance. As can be seen, most of the energy loss occurs between the TENG and the transmitting module, whereas the energy transmission efficiency between the transmitting module and the receiving module is very high and could be over 80%, depending strongly on the transmission distance.

**Table 2:** Performance summary for a MR-WTENG with the coil diameter of 10 cm. Inductance of both the coils is 62.5 μH, *C*_2_ = 140 pF.

Distance (cm)	2	5	10	15	20
Coupling coefficient *k*	0.3	0.12	0.032	9 × 10^−3^	2.5 × 10^−3^
Load *R*_*L*_ (Ω)	385	150	34	11	11
Harvested energy by TENG (µJ)	38.9	38.9	38.9	38.9	38.9
Energy at transmitter (µJ)	11.2	11.2	11.2	11.2	11.2
Energy at receiver (µJ)	9.9	8.176	3.44	0.448	4.8 × 10^−2^
Transmission efficiency by coupled circuit (%)	88.4	73	30.68	4.02	0.43
Transmission efficiency between TENG and load (%)	28.8	21	8.8	1.15	0.12

It should be emphasized that the energy transfer efficiency strongly depends on the coil diameter as well, and could be improved significantly if large coils are used. We built a coupling system with a coil diameter of 60 cm as shown in Fig. 3h, similar to the previous WPT system reported by Kursʼs group^22^, and investigated the energy transmission between the coils with the results shown in Fig. 3i. The energy transfer efficiency decreases quickly with increase in coil distance, but not as quickly as when 10 cm diameter coils were used. At a coil distance of 45 cm, an efficiency of ~45 % was obtained, much larger than that for the 10 cm diameter coil case. The efficiency of our MR-WTENG system is lower compared to that of the previous system^22^, possibly due to the low Q-factor and finite pulsed energy input for our system, whereas the previous system used a sinusoid voltage source^22^.

### Applications of MR-WTENG

**Fig. 4:**
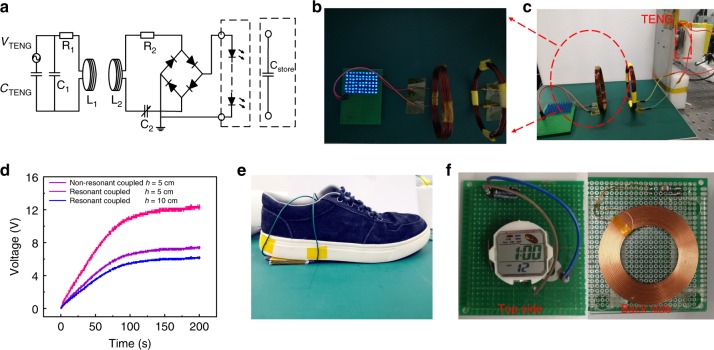
Applications of wireless power transfer. **a** Experimental setup for the wireless TENG and wireless power transfer. **b** A photo of the resonant-coupled transmitter and receiver coils. **c** A photo of the wirelessly lit up 70 40 mW-rated LEDs with high brightness. **d** The voltages of the storage capacitors as a function of charging time with the coil distance of 5 cm and 10 cm, respectively at the resonant-coupled and nonresonant-coupled states. The storage capacitor value is 15 µF for both the charging cases. **e** A photo of a shoe integrated with the MR-WTENG for harvesting walking energy. **f** The photo of a digital watch that could be wirelessly powered.

Three applications are demonstrated to show that MR-WTENG could wirelessly provide power: lighting up an LED array, charging up a capacitor, and wireless energy collection from a MR-WTENG integrated in a shoe to power a watch. Figure 4b, c shows the LEDs being wirelessly powered by the MR-WTENG. A 40 × 50 mm^2^ TENG was operated under 50 N contact force, 4 Hz contact frequency, and 4 mm separation distance. The parameters of the two coils and the capacitor *C*_1_ and *C*_2_ are shown in Table 1. The 70 blue LEDs rated at 40 mW were connected in series. The LEDs are lit up by the harvested energy wirelessly transmitted from the MR-WTENG. A video (Supplementary Movie 1) is provided in the Supplementary Information for details. Figure 4d shows the voltage of the storage capacitor (15 µF) in the receiving module as a function of time for the resonant-coupled and nonresonant-coupled cases, with the coil distances of 5 cm and 10 cm, respectively. The TENG was operated using a 50 N contact force, a 4 Hz contact frequency, and a 4 mm separation. It is shown that the resonant coupling can increase the charging speed and the saturated voltage for the capacitor significantly. For the resonant-coupled cases, the saturation voltage for a 5 cm coil distance is larger than that with a 10 cm coil distance. This is because the received voltage has a larger *V*_PP_ when the transmission distance is shorter as discussed above. A video (Supplementary Movie 2) to demonstrate this experiment is provided.

Figure 4e is a photo of a shoe integrated with a TENG, which could harvest walking or running energy. The transmitting coil was fixed inside one pocket of the trousers and connected to the TENG by wires, and the receiving module was placed outside the pocket. This will wirelessly receive and store the energy in a capacitor from the TENG continuously as the person walks. The receiving coil was connected to a capacitor through a rectifying circuit and the capacitor could provide power to a digital watch. The transmitting coil and energy harvesting module are shown in Fig. 4f. After about tens of steps walking, the digital watch could be powered up (shown in Supplementary Movie 3).

### Wireless chipless sensing applications

In the above analysis, it is shown that a maximum energy transfer efficiency of 73% could be achieved at a coupling distance of 5 cm for the resonant-coupled case, but it drops off rapidly with the increase of the coupling distance. However, if the system is not used for energy but instead for signal transmission, then the system could work efficiently for much longer distance signal transmission, making it particularly suitable for wireless sensing. Below, we will show the MR-WTENG system can be further modified for two types of wireless chipless (it is so called as the sensor system contains no microelectronic devices, microprocessor, and radio frequency (RF) module etc., but only passive capacitors and inductors) sensing applications.

**Fig. 5:**
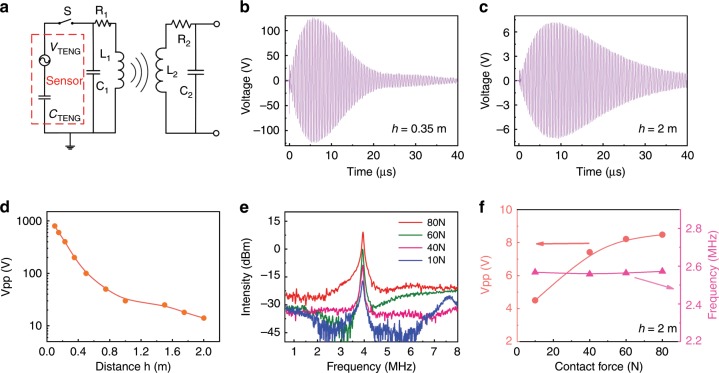
Wireless signal transmission for sensing application with TENG as the sensor. **a** System schematic of the wireless force sensor based on TENG. **b**, **c** are the received voltage signals with a coil distance of 0.35 m and 2 m, respectively. **d** The summary of the peak-to-peak voltage of the received signals as a function of coil distance. **e** The FFT spectrum of the received voltage signal under various contact forces. **f** The received peak-to-peak voltage and resonant frequency as a function of contact force with a 2 m coil distance. The inner and outer diameters of the coils are 21.5 cm and 27 cm, respectively.

The first type of self-powered wireless sensors was demonstrated by using the TENG as a sensor as well as the power source, with the configuration schematically shown in Fig. 5a. As previously shown, TENGs can be directly used as sensors for sensing force, pressure, vibration, acceleration, humidity, etc.^40,43,44^, as the amplitude of the output voltage of the TENGs strongly depends on these variables. Based on these previous examples, our system can be utilized as wireless chipless sensors for these same variables. In this case, the wirelessly transferred entity is the sensing signal voltage (amplitude and frequency) and the wireless transmission distance could be dramatically increased up to a few meters. Figure 5b, c are the receiving voltages measured by a probe with a 100 MΩ input impedance (equivalent to R_*L*_ = ∞, an open circuit) at a coil distance of 0.35m  and 2.0 m, respectively, and Fig. 5d is the summary of *V*_PP_ as a function of coil distance (note *C*_2_ was fine tuned to make the system working at the resonant-coupled state with the probe connected to the coil and the value of *C*_2_ is fixed during this sensing experiment.). For this experiment, a *C*_2_ capacitor of 45 pF (i.e., *C*_1_+*C*_TENG_ = 45 pF) and two coils with an inner diameter of 21.5 cm and an outer diameter of 27 cm were used. These have an inductance of 40 μH, resulting in an oscillating frequency of 4 MHz on the transmitter. The amplitude of the received oscillating signal decreases with increase of the distance, but at a much slower rate compared to those at the nonresonant-coupled state, and the received voltage waveform still has a maximum amplitude of *V*_PP_ = 14 V even at a transmission distance of 2.0 m with the system working at the resonant-coupled state. The energy transferred, however, is very small, close to zero, as the current through the oscilloscope is nearly zero. Further details of the transmitted voltage, *V*_PP_, as a function of TENG operating conditions (force, contact frequency, and spacer) and coupling distance are summarized in Supplementary Fig. 9 and Supplementary Note 9.

An example of a MR-WTENG based wireless force sensor is shown in Fig. 5e, f; Fig. 5e is the FFT spectra of the voltage signals obtained for different forces applied to the TENG, and Fig. 5f is the summary of resonant frequency and *V*_PP_ as a function of force at a coil distance of 2 m. The resonant frequency remains unchanged as it is determined by the transmitter (*C*_TENG_ is a constant with using the microswitch), while the signal amplitude increases with the force applied to the TENG.

**Fig. 6:**
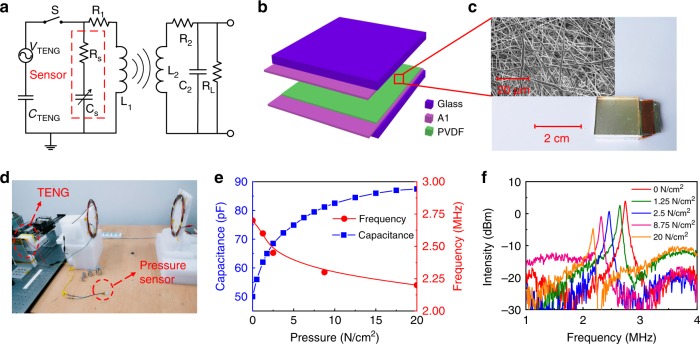
MR-WTENG-based wireless chipless sensor. **a** The configuration of the wireless chipless sensor system with the sensor integrated in the resonant circuit. **b**, **c** are the structure and photo of the electrospun PVDF nanofiber membrane-based capacitive-type force sensor. **d** The photo of the wireless sensing system. **e** The capacitance variation with different pressures applied to the sensor. **f** The FFT spectra received by the remote receiver at 2 m as a function of pressure applied on the sensor. The diameter of the two coils is 21.5/27 cm for this experiment, *C*_2_ = 82 pF and *R*_*L*_ = 3.3 kΩ.

Apart from the direct use of TENG as a wireless sensor, an impedance modulation circuit is another possible configuration for a fully self-powered wireless sensing application. As shown in Fig. 6, replacing the capacitor *C*_1_ in the resonant circuit with a capacitive sensor of any type with a capacitance value of *C*_S_, the resonant frequency of the system is now determined by the *L*_1_(*C*_S_+*C*_TENG_) circuit. A change in capacitance *C*_S_ of the sensor by external stimulation will result in a change of the resonant frequency of the transmitter. The TENG provides energy to generate the resonance signal containing the sensing information and transmits it wirelessly to the receiver. Figure 6a–c are the sensor system, photo, and capacitance variation of a capacitive pressure sensor integrated in the resonant circuit respectively. The sensor consists of an electrospun poly(vinylidene fluoride) (PVDF) nanofiber membrane sandwiched between two metal electrodes. Figure 6d is a photo of the pressure sensing setup with a weight added on the sensor to produce a static pressure. Before measurement, *C*_2_ and *R*_*L*_ were tuned to obtain the resonant state with the same frequency of the transmitter, and were fixed during sensing. For this experiment, a capacitance of *C*_2_ = 82 pF and a load of *R*_*L*_ = 3.3 kΩ were used. When a pressure is applied, the distance between the two electrodes of the pressure sensor varies, changing the capacitance of the sensor as shown in Fig. 6e. Varying *C*_S_ of the sensor results in small resonant frequency shifts of the system, and can be remotely detected by the receiver. Figure 6f is the FFT spectra of the voltage signals received by the receiver as a function of pressure applied, and clearly show a downward shift of frequency with the increase of pressure at a 2 m coil distance. The dependence of frequency on pressure is also shown in Fig. 6e. Note *C*_2_ can also be tuned to match the resonant frequency of the transmitter for sensing of each pressure change, but requires an automated matching circuit to achieve for this. In this case, the amplitude of the received signal would remain unchanged, different from that shown in Fig. 6f, where *C*_2_ and *R*_*L*_ were fixed once they were tuned at the resonant state.

Instead of using a capacitive type sensor, inductive sensors are also suitable for this type of wireless sensing based on the impedance modulation circuit. In this case, an inductive sensor with *L*_S_ could be used to replace the transmitter coil, *L*_1_, in the *L*_1_*C*_1_ resonant circuit.

As can be seen, these types of wireless sensor systems do not contain microelectronic components. They only contain the passive component inductors and capacitors, and require no energy conversion from one form to another, so that the energy utilization efficiency is very high, transmission distance is long, and it provides instantaneous response to stimulation. It is also worth emphasizing that these types of self-powered sensors are different from the so-called self-powered wireless sensors, in which the voltage signal is usually rectified, stored, and then regulated to power sensors, transmission chip, and other electronic components^45^.

## Discussion

In this work, we firstly demonstrated a MR-WTENG system. The pulsed voltage output of the TENG was modulated to provide an oscillating voltage that was wirelessly transmitted out and received by a receiver coil. The maximum energy transfer efficiency of up to 73% could be achieved for a 5 cm coil distance (10 cm coil diameter) when the system works at the resonant-coupled state, but the efficiency drops rapidly with increase of the coupling distance. Thus, the system is effective for wireless energy harvesting at relatively short distances. Besides as a wireless green energy source, the MR-WTENG system could also be used as a fully self-powered wireless chipless sensor. This is different to the so-called self-powered wireless sensors, where the voltage signals are usually rectified, stored as a harvested energy and then regulated to power sensors, transmission chip, and other electronic components. In our wireless chipless TENG sensors, the TENG oscillating signal (amplitude and resonant frequency) was utilized directly as the sensing parameter and as a power source, without using any power regulator and other integrated circuit chips. The sensor systems are very simple, particularly suitable for application in remote or harsh environments. It should also be emphasized that the resonant frequency of the chipless sensors can also be utilized as the identification (ID) of the sensors as we did in our previous work^34,35^, providing further sensing and ID information for this simple wireless system.

## Methods

### TENG fabrication and characterization

PDMS (184 Silicone Elastomer, Dow corning Co. Ltd.) and PA6 (Rhodia Ltd.) were used as the negative and positive tribomaterials respectively to make the TENG. PDMS prepolymer and cross-linker agent, and PA6 pellets were purchased from Dow Corning and Rhodia Ltd., respectively. Formic acid was obtained from Sinopharm Chemical Reagent Co., Ltd.

The PDMS fabrication process is as follows: first, the PDMS prepolymer and cross-linker agent were mixed in a weight ratio of 10:1 by mechanical stirring for 10 min and then degassed in a vacuum chamber for 50 min. Second, the mixture was spin-coated on clean glass substrates with a rotation speed of 500 rpm (rotations per minute) for 10 s, followed by 1200 rpm for 40 s to obtain the films. Then the PDMS-coated glass substrates were cured on a hot plate at 105 °C for 60 min. Finally, the dried PDMS films were peeled off from the glass substrates. The thickness of the PDMS films was about 100 μm in this work.

The synthesis of PA6 films followed the phase-inversion process developed by Soin et al.^46^. The doped solution was prepared using a 20 wt% solution in the solvent formic acid by continuously stirring at ~70 °C for 3 h. The prepared solution was deposited on a silicon wafer via a spin-coating process: initial spinning at 10 rpm for 5 s, and followed by 2000 rpm for 20 s. The coated substrate was then immersed immediately in an antisolvent bath kept at the desired quenching temperature of ~20 °C. The free-standing membranes with a thickness of 30 μm were then rinsed repeatedly with distilled water and left overnight to remove any residual solvent.

The PDMS and PA6 films were assembled into contact-mode TENG devices with two pieces of acrylic plates (55 mm × 55 mm × 2 mm) as the support substrates. Firstly, two pieces of nickel tape (40 × 55 mm^2^) were attached to two substrates respectively, as the conductive electrodes. Then the PDMS and PA6 films were attached to the other side of the nickel tapes.

A linear motor (H01-48 × 250) was utilized to control the cyclic contact force, frequency, and distance. The output voltage of the TENG was measured using an oscilloscope (Tektronix MDO3022).

### Wireless energy transmission TENG system

Two coils with a diameter of 10 cm were used in this experiment, both the coils have an inductance value of 62.5 μH. Both the two coils have a winding number *N*_1_ = 28, and the radius of the copper wire is *a*_1_ = 1 mm. The oscillating signal output of MR-WTENG was measured using an oscilloscope (Tektronix MDO3022).

### LED lighting up and capacitor charging up

Two coils with a diameter of 10 cm were used in this experiment, both the coils have an inductance value of 62.5 μH. Both the two coils have a winding number *N*_1_ = 28, and the radius of the copper wire is *a*_1_ = 1 mm. The circuit contains a bridge rectifier and 70 LEDs connected in series. For the charging capacitor application, the LEDs were replaced by a capacitor of 15 µF.

### Pressure sensor

An electrospun PVDF membrane with a thickness of about 200 µm was used as the dielectric material of the varying capacitor (pressure sensor). Commercial Al foil was used as the electrodes and sandwiched by two acrylic plate supports. The sensor has an area of 20 × 20 mm^2^.

## Supplementary information

Supplementary Information

Description of Additional Supplementary Files

Supplementary Movie 1

Supplementary Movie 2

Supplementary Movie 3

## Data Availability

The data that support the findings of this study are available from the corresponding author upon reasonable request.
