# Supplementary material for: Conjunction of triboelectric nanogenerator with induction coils as wireless power sources and self-powered wireless sensors
Source: Nat Commun. 2020 Jan 2;11:58. doi: 10.1038/s41467-019-13653-w (PMC6940365; doi:10.1038/s41467-019-13653-w)
Supplement: Supplementary file 1 — Supplementary Information [file 41467_2019_13653_MOESM1_ESM.pdf]

Supplementary Information for

Conjunction of triboelectric nanogenerator with induction coils as wireless power sources and self-powered wireless sensors

Zhang *et al.*

Supplementary Note 1.

General performance of the PA6/PDMS TENG.

The standalone TENG (without the synchronized microswitch and LC circuit connected) was characterized under various conditions to verify the performance of the nanogenerator fabricated. The performance of the TENG with a dimension of 40 × 50 mm^2 at different contact forces, spacer distances, contact frequencies and load resistances are summarized in Supplementary Fig. 1. The maximum peak power output is 18.9 mW (9.45 W/m^2) at a load resistance of ~50 MΩ.

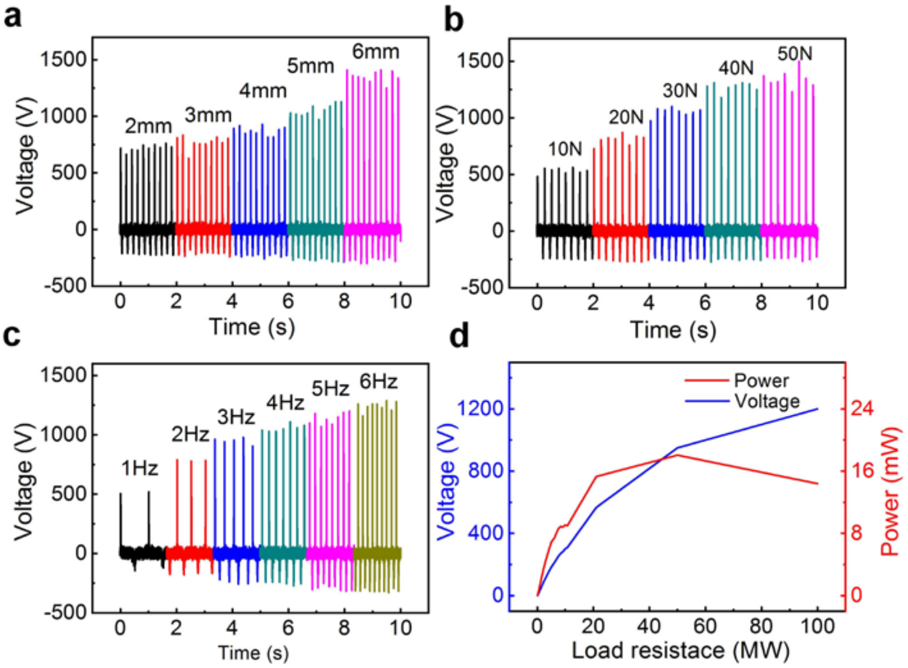

Supplementary Figure 1. Performance of PA6/PDMS TENG. a Open-circuit voltage of the TENG under different contact forces, showing the increasing trend of outputs. **b** The open-circuit voltage with different spacer distances, showing an increase with the increase of spacer distance. **c** The influence of the contact frequency on the open-circuit voltage, which increases when impact frequency rises from 1 Hz to 6 Hz. **d** The output voltage and the instantaneous peak power of the PA6/PDMS TENG as a function of load resistance.

Supplementary Note 2.

Schematic and theoretical analysis of the contact-separation mode TENG.

A simple contact-separation mode triboelectric nanogenerator has a structure schematically shown in Supplementary Fig. 2, consisting of a positive tribo-material and a negative tribo-material. The metal film on surfaces of the two triboelectric materials form the electrodes.

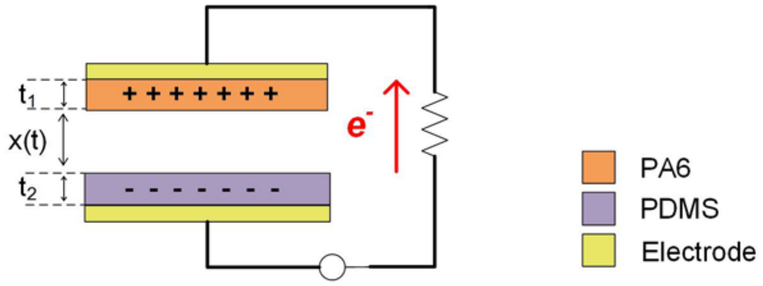

Supplementary Figure 2. Schematic of the contact-separation mode TENG. The TENG consists of a positive triboelectric plate with a thickness of t_1, and a negative triboelectric plate with a thickness of t_2. x(t) is the distance between the two tribo-plates and varies under dynamic operation.

The contact-separation mode TENG can be regarded as a pulse voltage source connected with a capacitor in series. At the maximum separation distance, its capacitance and voltage value are determined by the following formulas,[1]

$$C_{TENG} = \frac{\varepsilon_0 S}{t_0 + t_{max}} \tag{1}$$

$$V_{TENG} = \frac{\sigma t_{max}}{\varepsilon_0} \tag{2}$$

where σ, t_{max}, ε_0 and S represent the surface charge density, the maximum distance between the two friction layers, the vacuum dielectric constant and the surface contact area, respectively. t_0 is the effective thickness of TENG and is defined as:

$$t_0 = \frac{t_1}{\varepsilon_{r1}} + \frac{t_2}{\varepsilon_{r2}} \tag{3}$$

where t_1 and t_2 are the thicknesses of the positive and negative tribo-materials, and ε_{r1} and ε_{r2} are the relative dielectric constants of the tribo-materials, respectively.

Supplementary Note 3.

Working principle of the bi-contact microswitch.

To increase the output power and reduce the output resistance of the TENG, a synchronized microswitch was integrated with the TENG[1-3]. Different from the previous work[2], a bi-contact microswitch was used in this work as it is able to utilize both the positive and negative voltage pulses of a TENG, so that to increase the energy harvesting efficiency. The schematic drawing and working principle of the microswitch are shown in Supplementary Fig. 3. The microswitch

is switched on after the two tribo-plates are at the close state (the two plates contact each other) or at the open state (the two plates are at the maximum separation distance), then the energy generated by the TENG is transferred to the resonant circuit.

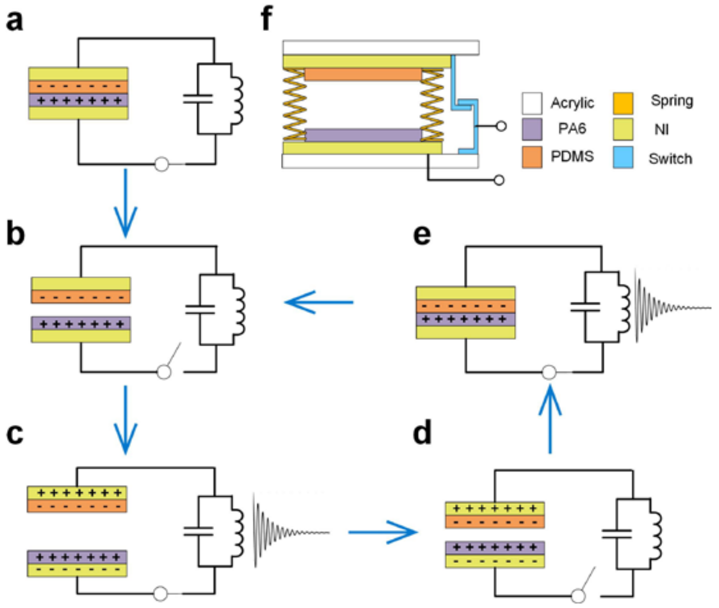

Supplementary Figure 3. A flow chart of the TENG with a synchronized microswitch. a- e The synchronized movement of the microswitch with the movement of the TENG plate. **f** Schematic of the TENG with a integrated microswitch.

Supplementary Note 4.

Theoretical analysis of MR-WTENG system.

From the Kirchoff's Law, we can obtain the following differential equations from the equivalent circuit,

$$L_1\frac{d^2i_1}{dt^2} - M\frac{d^2i_2}{dt^2} + R_1\frac{di_1}{dt} + \frac{i_1}{C_1+C_{TENG}} = U_0\delta(t) \tag{4}$$

$$L_2\frac{d^2i_2}{dt^2} - M\frac{d^2i_1}{dt^2} + (R_2 + R_L)\frac{di_2}{dt} + \frac{i_2}{C_2} = 0 \tag{5}$$

$$u_1 = L_1\frac{di_1}{dt} \tag{6}$$

$$u_2 = R_L i_2 \tag{7}$$

Here i_1 and i_2 are the currents in Coil 1 and Coil 2, respectively, M is the mutual inductance between the two coils, C_{TENG} is the capacitance of the TENG shown above. The currents and voltages can be solved using the Laplace transform as follow,

$$L_1 s^2 I_1(s) - M s^2 I_2(s) + R_1 s I_1(s) + \frac{I_1(s)}{C_1 + C_{TENG}} = U_0 \tag{8}$$

$$L_2 s^2 I_2(s) - M s^2 I_1(s) + (R_2 + R_L) s I_2(s) + \frac{I_2(s)}{C_2} = 0 \tag{9}$$

$$U_1(s) = L_1 s I_1(s) \tag{10}$$

$$U_2(s) = R_L I_2(s) \tag{11}$$

From Supplementary Eq. 8 to 11, we obtain

$$U_2(s) = \frac{R_L M U_0}{(L_1 L_2 - M^2)\mathrm{s}^4 + (L_2 R_1 + L_1(R_2 + \mathrm{R_L}))s^3 + (\frac{L_2}{C_1 + C_{TENG}} + \frac{L_1}{C_2} + R_1(R_2 + \mathrm{R_L}))s^2 + (\frac{R_2 + R_L}{C_1 + C_{TENG}} + \frac{R_1}{C_2})s + \frac{1}{(C_1 + C_{TENG})C_2}} \tag{12}$$

If we let

$$F(s) = (L_1 L_2 - M^2)\mathrm{s}^4 + (L_2 R_1 + L_1(R_2 + \mathrm{R_L}))s^3 + (\frac{L_2}{C_1 + C_{TENG}} + \frac{L_1}{C_2} + R_1(R_2 + \mathrm{R_L}))s^2 + (\frac{R_2 + R_L}{C_1 + C_{TENG}} + \frac{R_1}{C_2})s + \frac{1}{(C_1 + C_{TENG})C_2} \tag{13}$$

then it can be proven that the function F(s) has no real solution under the condition that

$$C_1 + C_{TENG} << \frac{4L_1}{R_1^2}, C_2 << \frac{4L_2}{(R_2 + \mathrm{R_L})^2}$$

For this case, Supplementary Eq. 12 becomes

$$U_2(s) = \frac{k_1(s - \alpha_1)}{(s - \alpha_1)^2 + \beta_1^2} + \frac{k_2 \beta_1}{(s - \alpha_1)^2 + \beta_1^2} + \frac{k_3(s - \alpha_2)}{(s - \alpha_2)^2 + \beta_2^2} + \frac{k_4 \beta_2}{(s - \alpha_2)^2 + \beta_2^2} \tag{14}$$

where α_1, α_2, β_1 and β_2 are determined by the values of L, C, R, M and U_0 of the TENG. Making the inverse Laplace transform yields

$$u_2(t) = k_1 e^{\alpha_1 t} \cos(\omega_1 t) + k_2 e^{\alpha_1 t} \sin(\omega_1 t) + k_3 e^{\alpha_2 t} \cos(\omega_2 t) + k_4 e^{\alpha_2 t} \sin(\omega_2 t) \tag{15}$$

Taking the triangular identity transformation, we obtain

$$u_2(t) = k_5 e^{\alpha_1 t} \sin(\omega_1 t + \varphi_1) + k_6 e^{\alpha_2 t} \sin(\omega_2 t + \varphi_2) \tag{16}$$

Similarly,

$$u_1(t) = k_7 e^{\alpha_1 t} \sin(\omega_1 t + \varphi_3) + k_8 e^{\alpha_2 t} \sin(\omega_2 t + \varphi_4) \tag{17}$$

The solution of the undetermined coefficients in Supplementary Eq. 16 and 17 is very complicated, but we can use MATLAB to find specific values.

With the parameters shown in Table 1 (C_2 is a variable), all the coefficients can be solved and the relationship of the undetermined coefficient of Eq. 6 with C_2 is shown in Supplementary Fig. 4.

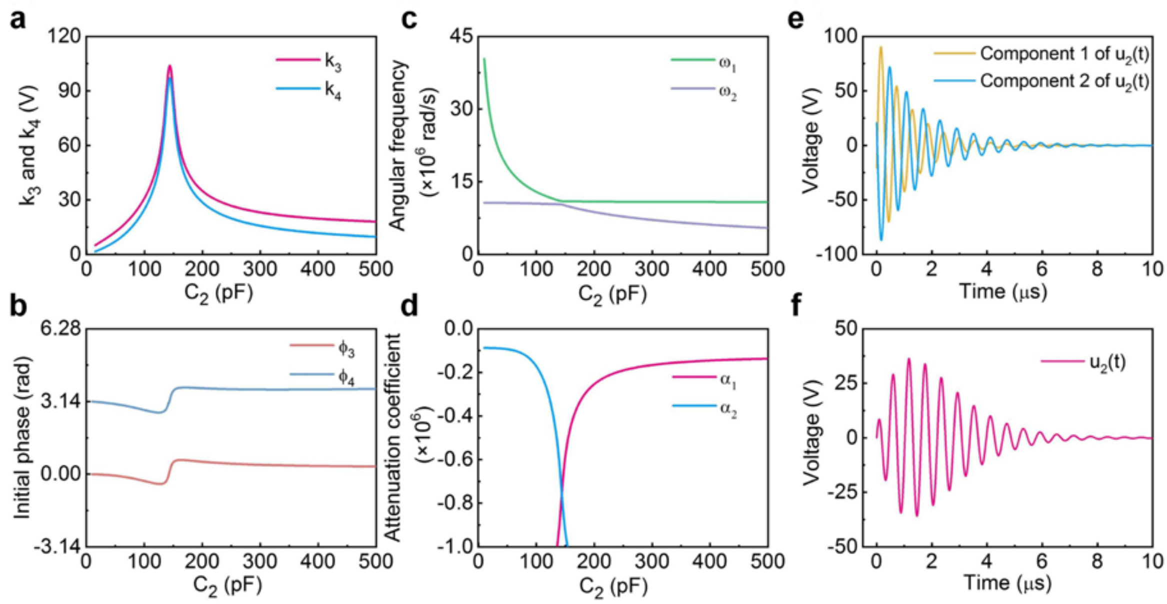

Supplementary Figure 4. Theoretical analysis of pulsed voltage transmission. **a, b, c** and **d** show the relationship between the coefficients (k, φ, ω, α of Eq. (6)) and tuning capacitor C_2. **f** The received signal at the resonant-coupled state was obtained by superimposing two signal components in **e**. For the analysis, the distance between the two coils was fixed at 5 cm and the load resistance was fixed at R_L=150 Ω.

Supplementary Note 5.

Formation of waveform with envelop of the received signal.

Theoretical analysis has been conducted to understand the received waveform with multiple envelops for the system working at a load resistance smaller than the optimal load. As stated, there are two oscillation components with frequencies slightly different from each other and the phase difference of π. Supplementary Fig. 5a shows the waveforms of the two components obtained with the conditions as those for Fig. 3a. When these two waveforms are superimposed, it produces a waveform shown in Supplementary Fig. 5b with multiple envelops, similar to what shown in Fig. 3a, clearly indicating that the load resistance would affect the energy transmission efficiency.

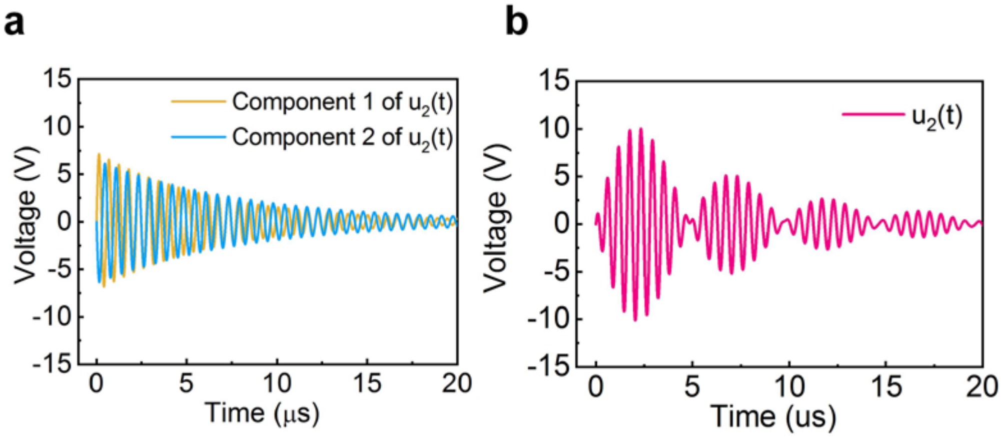

Supplementary Figure 5. Formation of waveform with multiple envelops. a The waveforms of the two oscillating components of the received signal at the resonant-coupled state obtained by theoretical analysis with different frequencies and phase difference of π, and **b** was obtained by superimposing the two signal components in **a**, showing a waveform with multiple envelops, similar to those experimentally obtained. Here R_L=28 Ω.

Supplementary Note 6.

Configuration of MR-WTENG.

Schematic drawing of the magnetically coupled wireless energy transmission is shown in Supplementary Fig. 6a, while a photo of the system in Supplementary Fig. 6b. Shown in the figure are the coupling coils with an inner diameter of 10 cm.

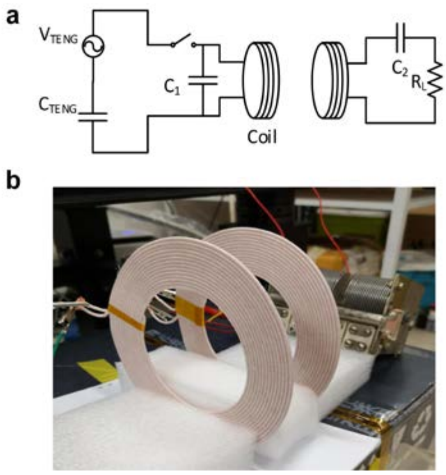

Supplementary Figure 6. MR-WTENG system configuration and photo. a Schematic and equivalent circuit of the proposed magnetically resonance coupled energy transferring TENG system. **b** The photo of the MR-WTENG system for wireless energy transferring.

Supplementary Note 7.

Mutual inductance.

The wireless energy transmission system adopts the structures of two air cored coils as shown in Supplementary Fig. 7a, one of which is the energy transmitting coil L_1 and the other is the energy receiving coil L_2. The radius of the two coils are r_1 and r_2, respectively, and the distance is h. Based on the principle of electromagnetic field, the mutual inductance between the two inductor coils is calculated using the Norman formula:

$$M = \frac{\mu_0 N_1 N_2}{4\pi} \oint_{l_1} \oint_{l_2} \frac{d\vec{l_1} d\vec{l_2}}{r} \tag{18}$$

where l_1, l_2 represent the circumference of the loops 1 and 2 respectively, dl_1, dl_2 are two-line elements taken on the two coil loops l_1, l_2, and the angle of the two-line elements is described by the polar coordinates θ, which is also the angle between the two-line vector. When the two coils are circular and the number of turns is N_1 and N_2, we obtain the following formula.

$$d\vec{l}_2 \cdot d\vec{l}_1 = r_1 r_2 \cos\theta \, d\theta d\phi$$

$$r = \sqrt{{r_1}^2 + {r_1}^2 - 2r_1 \cdot r_2 \cos\theta + h^2} \tag{19}$$

Inserting Supplementary Eq. 19 in 18, we obtain,

$$M = \frac{\mu_0 N_1 N_2}{4\pi} \int_0^{2\pi} \int_0^{2\pi} \frac{r_1 r_2 \cos\theta}{r} d\theta d\phi \tag{20}$$

Using variable substitution and elliptic integral, then

$$M = \mu_0 N_1 N_2 \frac{\sqrt{r_1 r_2}}{b} [(2 - b^2)K(b) - 2E(b)], \; b = \sqrt{\frac{4 r_1 r_2}{(r_1 + r_2)^2 + h^2}} \tag{21}$$

For large M for high energy transmission, let $\lambda = r_1 / r_2$, $\mu = h / r_2$, and substitute them into Supplementary Eq. 21,

$$M = \mu_0 N_1 N_2 r_2 \sqrt{(1+\lambda)^2 + \mu^2} [(1 - b^2/2)K(b) - E(b)], \; b = \sqrt{\frac{4\lambda}{(1+\lambda)^2 + \mu^2}} \tag{22}$$

The expression for the mutual inductance contains the first and second types of fully elliptic integrals K(b) and E(b), and their values can be obtained using the mathematic manual or the elliptic integral function in MATLAB. Supplementary Fig. 7b shows dependence of the mutual inductance on coil distance with the radius ratio of the two coils as a variable, showing that the mutual inductance M decreases as the distance increases. The coupling coefficient is at the maximum when the ratio is λ=1 and the distance between the two coils is 0. Supplementary Fig. 7c shows the variation of the mutual inductance as a function of coil ratio with the distance

between the two coils as a variable. The results show that the maximum mutual inductance, *M*, always occurs at the coil radius ratio of λ=1, and M decreases rapidly when the coil radius ratio deviates from the unit. This is the reason we choose the transmitter and receiver coils with the same diameters for better energy transmission.

The mutual inductance coefficient K is more convenient for characterizing the energy transmission efficiency in the resonant coupled energy transmission system. The coupling coefficient through the mutual inductance is given by the following formula,[4]

$$K = \frac{M}{\sqrt{L_1 \cdot L_2}} \tag{23}$$

This reflects the tightness of the coupling of the two coils more accurately. The transmitting coil L_1 used in our experiment has a coil diameter d_1=10 cm, a winding number N_1 = 18, and a copper wire cross-sectional radius a_1 = 1 mm. The parameter of the coil L_2 is the same as L_1. Supplementary Fig. 7d shows the coupling coefficient K of the coils used for the experiment as a function of distance between the two coils, showing rapid decrease of the coefficient as the distance increases. The coupling coefficient is 0.1 when the distance is equal to the radius of the coils.

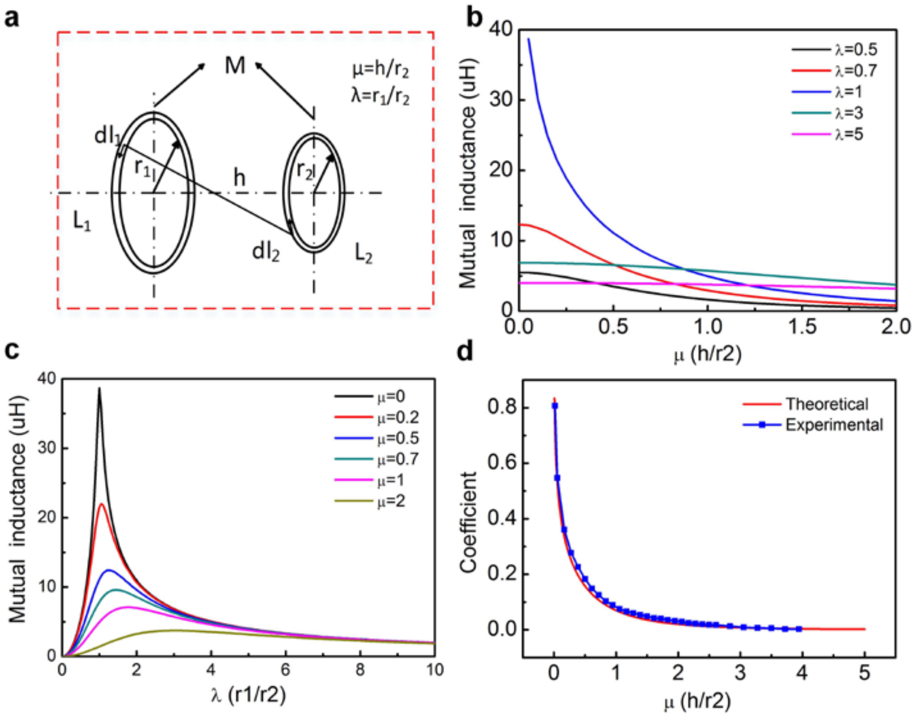

Supplementary Figure 7. Mutual inductance between the two coaxial coils. a Model of coaxial coil mutual inductance. **b** and **c** the mutual inductance changes with the radius and distance of the two

coils, and the independent variable is given by the following expression: $\lambda=r_1/r_2$, $\mu=h/r_2$. **d** The coefficient of mutual inductance varies with distance at $\lambda=1$ and $\mu=1$.

Supplementary Note 8.

Total transferred energy influenced by abnormal high frequency jitter.

It is clear that the high frequency abnormal jittering signal still exists at the beginning of the received resonant signal as shown in Supplementary Fig. 8b. This leads to a difference in energy transferred of the first half period (between two black dashed lines). The energy transferred by the high frequency component is about 46 nJ and 25 nJ for experimental and theoretical results, respectively. This is very small part (less than 1%) of the total transferred energy as shown in Supplementary Fig. 8a, which is ~8.2 μJ for both the experimental and theoretical results. Thus, the abnormal jittering signal should be considered negligible under the current experimental setup for analysis.

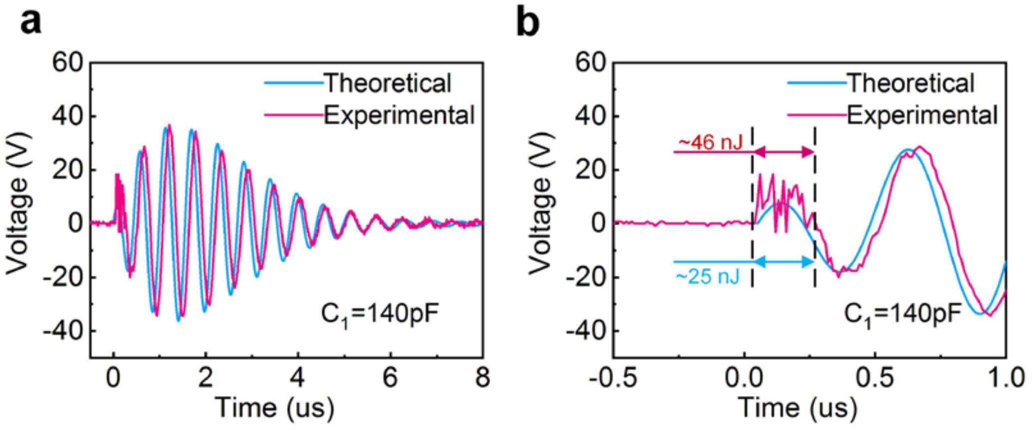

Supplementary Figure 8. Influence of abnormal jitter on the received voltage waveform. a The overall and **b** the detailed voltage waveforms including theoretical and experimental energy results, demonstrating that the jitter can be considered negligible due to its small signal.

Supplementary Note 9.

Characterisation of MR-WTENG as a signal transmitter.

Summary of V_{PP} of the transmitted voltages for the 21.5/27cm coils as a function of TENG operating conditions (force, contact frequency and spacer) as well as the coil distance are shown Supplementary Fig. 9.

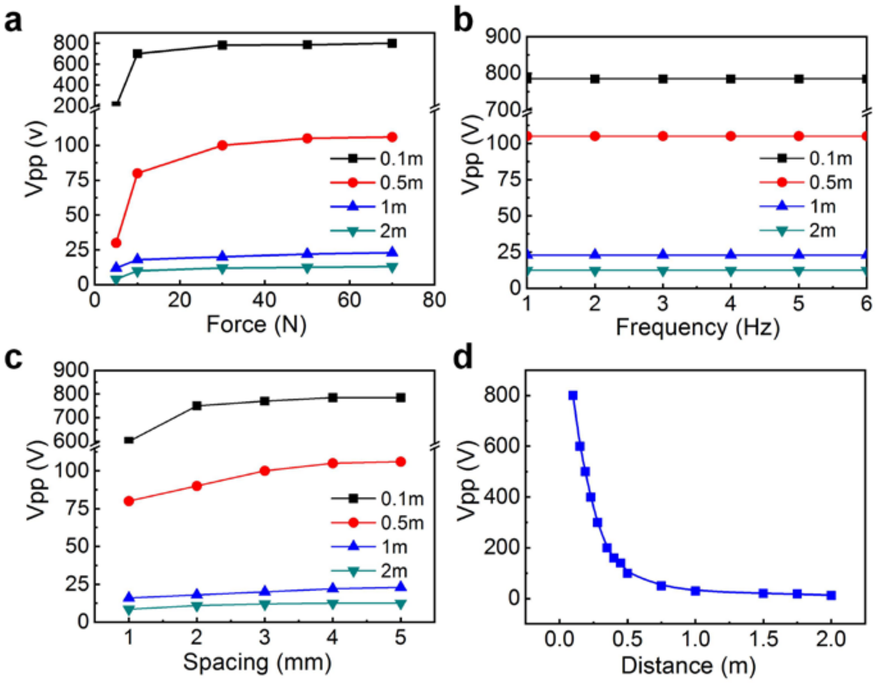

Supplementary Figure 9. Peak-to-peak voltage as a function of operation condition. The Vpp of the received voltage with different distance between the two coils under various forces **a** with contact frequency and spacing distance fixed at 4 Hz and 4mm, respectively; under various contact forces **b** with contacted force and spacing distance fixed at 50N and 4 mm, respectively; and various spacing distance between the two tribo-plates **c** with the contacted force and frequency fixed at 50 N and 4 Hz. The Vpp of the received voltage decreases rapidly with the increase of the coil distance **d** under TENG working condition at 50 N contact force, 4 Hz contact frequency and 4 mm spacing distance. The TENG size is 40 × 50 mm^2, and the coil diameter is 21.5/27 cm.

Supplementary References

1. Vasandani, P., Gattu, B., Mao, Z. H., Jia, W.Y.; Sun M.G., Using a Synchronous Switch to Enhance Output Performance of Triboelectric Nanogenerators . *Nano Energy* , **43** 210-228 (2017).
2. Chen, J.K., Xuan, W.P., Zhao, P.F., Farooq, U., Ding P., Yin, W.; Jin H., Wang, X.Z.; Fu, Y.Q.; Dong, S.R, Triboelectric effect based instantaneous self-powered wireless sensing with self-determined identity. *Nano Energy*, ***51***, 1–9 (2018).

3. Yin, W.L, Xie, Y.D, Long, J., Zhao, P.F., Chen, J.K, Luo, J.K., Wang, X.Z., Dong, S.R. A self-power-transmission and non-contact-reception keyboard based on a novel resonant triboelectric nanogenerator (R-TENG). *Nano Energy*, ***50***,16-24 (2018).
4. Frivaldsky, M., Piri, M., Jaros, V. Verification of a mutual inductance calculation between two helical coils, *International Scientific Conference on Electric Power Engineering*, 712-717, (2015).
