# Supplementary material for: Conjunction of triboelectric nanogenerator with induction coils as wireless power sources and self-powered wireless sensors
Source: Nat Commun. 2020 Jan 2;11:58. doi: 10.1038/s41467-019-13653-w (PMC6940365; doi:10.1038/s41467-019-13653-w)
Supplement: Supplementary file 2 — Description of Additional Supplementary Files [file 41467_2019_13653_MOESM2_ESM.pdf]

Description of Additional Supplementary Files

File name: Supplementary Movie 1

Description: MR-WTENG lit up LEDs wirelessly.

File name: Supplementary Movie 2

Description: MR-WTENG charges up a capacitor wirelessly.

File name: Supplementary Movie 3

Description: MR-WTENG powers a digital watch wirelessly.
